# In a bind: a quality improvement project for reducing fluoroquinolones drug–drug interactions with multivalent cations

**DOI:** 10.1093/jacamr/dlag037

**Published:** 2026-03-25

**Authors:** Emily A Siegrist, Steven J Pan, Maria Alkozah, Danh Doan, Bryan P White

**Affiliations:** Department of Pharmacy, OU Health, Oklahoma City, OK, USA; Biostatistics and Epidemiology, Hudson College of Public Health, University of Oklahoma Health Sciences Center, Oklahoma City, OK, USA; Infectious Diseases Section, Department of Medicine, University of Oklahoma Health Sciences Center, Oklahoma City, OK, USA; Department of Pharmacy, OU Health, Oklahoma City, OK, USA; Department of Pharmacy, OU Health, Oklahoma City, OK, USA

## Abstract

**Introduction:**

Fluoroquinolone (FQ) co-administration with multivalent cations can decrease the bioavailability of the FQ significantly. This interaction is an independent risk factor for FQ resistance, which is a concern as more clinical trials and guidelines support routine use of oral antimicrobials.

**Methods:**

We examined the incidence of FQ drug–drug interactions (DDIs) in a quality improvement study after implementation of a bundle of interventions including nursing education, updated medication administration record (MAR) comments and new administration times.

**Results:**

In the subgroup of patients (*n* = 101) who had a multivalent cation administered on the same day as a FQ, co-administration within 2 hours of the FQ was higher in the pre-intervention group as compared to the post-intervention group (62% versus 22%, *P* < 0.001).

**Conclusion:**

The bundle of interventions decreased but did not eliminate the interaction. With the interaction as an independent risk factor for resistance and increasing use of oral antibiotics, stewardship programmes should consider implementing similar interventions.

## Introduction

Fluroquinolones have been shown to have a decrease in bioavailability of up to 88% when co-administered with multivalent cations such as aluminium, iron, and magnesium.^1^ Drug–drug interactions (DDIs) between fluoroquinolones (FQs) and multivalent cations are common in the inpatient setting occurring in up to 72% of FQ courses.^2,3^ The decreased bioavailability of FQs is an independent risk factor for resistance development.^4–6^ Various interventions including education, direct pharmacist intervention after order verification and changing the standard time of FQ administration have been evaluated to decrease the incidence of FQ and cation DDIs.^4,5,7–9^ These studies often added single interventions to other strategies previously in place as opposed to looking at a bundle of interventions. Few trials have been conducted since the FQ prophylaxis for neutropenic fever (NF) in high-risk cancer patients became standard of care in 2010, which may have increased inpatient FQ use and potential DDIs.^10^ Newer electronic medical records (EMRs) have alerts for ordering providers about the FQ cation interaction, but with alert fatigue, their impact is unknown.^11^ There is a paucity of data about management and prevention of FQ-multivalent cation DDIs in modern EMRs. This study’s aim was to evaluate the effectiveness of a quality improvement project in reducing the incidence of FQ-multivalent cation DDIs in modern EMRs since the change in practice with FQ prophylaxis for NF.

## Methods

This is a single centre, retrospective, pre- and post-intervention, IRB approved study on the impact of a bundle of interventions to decrease the incidence of oral FQ and multivalent cation interactions. Before the intervention, providers and pharmacists saw alerts for the FQ cation interaction on order entry and verification, respectively. There were no comments on the medication administration record (MAR) or standard dosing times for FQs. The FQ and cation intervention had not been addressed in antimicrobial stewardship education provided annually to nursing, providers or pharmacists.

Between November 2024 and January 2024, we implemented a bundle of interventions at a single hospital system targeted towards reducing our oral multivalent cation administration within 2 hours before or after administration of formulary oral FQs, ciprofloxacin and levofloxacin. These interventions included a passive administration comment visible to nursing on the MAR when cations and FQs were ordered concomitantly (Figures S1 and S2, available as Supplementary data at *JAC-AMR* Online), nursing education handouts and alteration of standard time of FQ administration. FQ daily orders were changed to 0600 and twice daily changed to 0600 and 1800. Standard administration time for other orders is 0900 and 2100. Nursing education handouts were disseminated through nursing leadership and included information on the bundle. The EMR used was EPIC (Madison, WI, USA). We collected 1 month of oral FQ and oral cation administrations pre-intervention (September 2024) and 1 month post-intervention (February 2025). Patients were included if they had at least one administration of an oral FQ during the study period. Patients were identified using a slicer/dicer report in EPIC (Madison, WI, USA) for oral FQ administration. All included patients were manually reviewed for co-administration, defined from MAR administration time. No formulary updates or changes in stewardship practices related to FQs occurred during the study period. Concurrent tube feeds were defined as an order for tube feeding on the same day that oral FQs were administered. The primary outcome was the incidence of co-administration of an oral cation within 2 hours of an oral FQ for patients administered oral cations on the same day as oral FQs before and after the bundle of interventions. Secondary outcomes include the primary outcome in the subgroup of patients receiving FQs for NF prophylaxis and the overall rate of oral FQ and oral cation interaction between the pre- and post-intervention groups.

Results were summarized descriptively among the overall cohort and pre- and post-intervention groups. Differences between groups were assessed for statistical significance using the chi-square test or Fisher’s exact test for categorical data and the *t*-test or Wilcoxon rank sum test for continuous data as appropriate. Data management and analyses were conducted in R v,4.4.0.

## Results

Three hundred and ten patients were included in the study. The mean age was 53 years (SD 20). Levofloxacin (63%, *n* = 194) was the most common FQ administered (Table 1). The most common indication for FQ use was NF prophylaxis (34%, *n* = 104). The median inpatient duration of therapy was 3 days (IQR 1–7). The most common provider group prescribing FQ in the whole cohort was Hospital Medicine (30%, *n* = 94).

**Table 1.** Baseline characteristics

Characteristic	Overall (*n* = 310)^a^	Pre-intervention (*n* = 149)^a^	Post-intervention (*n* = 161)^a^	*P*
Age	53 (20)	53 (22)	54 (19)	>0.9
Gender				0.8
Female	154 (50%)	73 (49%)	81 (50%)	
Male	156 (50%)	76 (51%)	80 (50%)	
Primary team				
Hospitalist	94 (30%)	54 (36%)	40 (25%)	
Oncology/BMT	48 (15%)	20 (13%)	28 (17%)	
Other	42 (13.5%)	17 (11.4%)	25 (15.6%)	
IM	35 (11%)	15 (10%)	20 (12%)	
ER	30 (9.7%)	14 (9.4%)	16 (9.9%)	
Surgery	29 (9.4%)	11 (7.4%)	18 (11%)	
Gyn/oncology	16 (5.2%)	6 (4.0%)	10 (6.2%)	
Paediatrics	16 (5.2%)	12 (8.1%)	4 (2.5%)	
FQ				0.6
Levofloxacin	194 (63%)	91 (61%)	103 (64%)	
Ciprofloxacin	116 (37%)	58 (39%)	58 (36%)	
Indication				
NF prophylaxis	104 (34%)	50 (34%)	54 (34%)	
Urinary tract infection	57 (18%)	32 (21%)	25 (16%)	
Pneumonia	49 (16%)	21 (14%)	28 (18%)	
Intra-abdominal infection	30 (9.7%)	16 (11%)	14 (8.8%)	
Other	27 (8.7%)	6 (4.0%)	21 (13%)	
Osteomyelitis	22 (7.1%)	11 (7.4%)	11 (6.9%)	
Skin and soft tissue infection	9 (2.9%)	8 (5.4%)	1 (0.6%)	
Bacteraemia	6 (1.9%)	3 (2.0%)	3 (1.9%)	
Cirrhosis/spontaneous bacterial peritonitis prophylaxis	4 (1.3%)	1 (0.7%)	3 (1.9%)	
Surgical prophylaxis	1 (0.3%)	1 (0.7%)	0 (0%)	
FQ duration (days)	3.0 (1.0, 7.0)	2.0 (1.0, 5.0)	3.0 (1.0, 8.0)	0.5

^a^Mean (SD); *n* (%); Median (Q1, Q3).

We included 149 patients in the pre-intervention group and 161 patients in the post-intervention group. Forty-three percent of patients (*n* = 101) had oral multivalent cations administered on the same day as an oral FQ. In the subgroup of patients who had a multivalent cation administered on the same day as a FQ, co-administration within 2 hours of the FQ was higher in the pre-intervention group as compared to the post-intervention group (62% versus 22%, *P* < 0.001). Multivalent cations were administered on the same day as oral FQs in more patients in the post-intervention cohort (25% versus 40%, *P* = 0.005) (see Table 2). The most common concurrent multivalent cation was magnesium (13%). The median duration of overlap between FQs and multivalent cations co-administered within 2 hours was 2 days in the pre-intervention group (IQR 2–4.5) and 2 days in the post group (IQR 1–7).

**Table 2.** Cation data and intervention outcomes

Characteristic	Overall (*n* = 310)^a^	Pre-intervention (*n* = 149)^a^	Post-intervention (*n* = 161)^a^	*P*
Multivalent Cations administered on same day of FQ administration	101 (33%)	37 (25%)	64 (40%)	0.005
Magnesium	40 (13%)	14 (9.4%)	26 (16%)	0.076
Calcium	18 (5.8%)	5 (3.4%)	13 (8.1%)	0.076
Iron	16 (5.2%)	4 (2.7%)	12 (7.5%)	0.058
Aluminium	9 (2.9%)	1 (0.7%)	8 (5.0%)	0.038
Sevelamer	5 (1.6%)	2 (1.3%)	3 (1.9%)	>0.9
Carafate	2 (0.6%)	2 (1.3%)	0 (0%)	0.2
Multivitamin	2 (0.6%)	0 (0%)	2 (1.2%)	0.5
Zinc	1 (0.3%)	1 (0.7%)	0 (0%)	0.5
Co-administration within 2 hours^b^	37/101 (37%)	23/37 (62%)	14/64 (22%)	<0.001
Duration of FQ and cation overlap (days)	2.0 (1.0, 5.0)	2.0 (2.0, 5.0)	2.0 (1.0, 7.0)	0.7
Concurrent tube feeds	14 (4.5%)	7 (4.7%)	7 (4.3%)	0.9
Duration of FQ and tube feeds overlap (days)	2.0 (2.0, 5.0)	2.0 (2.0, 5.0)	3.0 (2.0, 8.0)	0.2

^a^Mean (SD); *n* (%); Median (Q1, Q3).

^b^Out of the 101 patients that got multivalent cations administered on the same day as an oral FQ.

In the NF subgroup (*n* = 104), the duration of FQ therapy was 8 days (IQR 3–14). Forty-one percent of patients had concurrent multivalent cations. The duration of FQ and cation overlap was 3 days (IQR 2–14). Co-administration within 2 hours was numerically lower in the post-intervention cohort, but was not statistically significant (24% versus 13%, *P* = 0.15).

## Discussion

The aim of this study was to evaluate the effectiveness of a quality improvement bundled intervention in reducing the incidence of FQ-multivalent cation DDIs. We showed a significant decrease in the percentage of multivalent cations administered within 2 hours of FQs using our interventions that included a change to the standard time of FQ administration and nursing education. Although the incidence of the DDI was reduced, it was not eliminated.

Similar to the study by Briceland published in 2009, we showed that our combination of education, administration timing change and MAR comments led to a decrease in the co-administration of FQs within 2 hours of and cations.^8^ We could replicate their results in a larger patient population (80 patients versus 310) and across a whole hospital as compared to specific units.^8^ Similar to Zimmerman, the impact of the intervention decreased but did not totally eliminate co-administration.^9^

FQ use for NF prophylaxis is associated with a significant decrease in mortality (up to 62% relative risk reduction).^7^ Patients receiving quinolones for NF prophylaxis were on therapy for a longer duration (8 versus 3 days) as compared to the total cohort and had similar overall rates of cation concurrent administration (41% versus 33%). There was a numerical decrease in FQ and cation concurrent administration in this subgroup, but it was not statistically significant, probably due to smaller sample size. With the benefit of FQ prophylaxis for NF prophylaxis, future research should examine new interventions to decrease the frequency of DDI in patients hospitalized who require NF prophylaxis.

The 2016 IDSA guidelines on antimicrobial stewardship, only had five strong recommendations (including increasing the use of oral antibiotics).^12^ With high quality trials of oral therapy with high systemic exposures including FQs showing no difference in outcomes as compared to IV therapy for disseminated infections, optimizing the use of oral antibiotics should be a priority for stewardship programmes.^13,14^ We have shown a bundle of interventions to optimize the use of FQ antibiotics that should be considered for implementation in other hospital systems to refine oral antimicrobial use.

We learned that it is difficult to coordinate a multi-intervention bundle to be all implemented at the same time. The nursing education that we provided was a handout delivered by nursing managers at huddles, and this method of disseminating education may be less effective than online modules or in person education from stewardship staff. More involvement and communication with our nursing colleagues would have likely made our education more effective. With the bundle not fully eliminating co-administration, stewardship programmes could consider having an alert in their clinical decision support to this interaction to make interventions after verification. We are planning to implement a similar bundle with integrase inhibitors.

Limitations of our study include the short pre- and post-intervention follow up time and single centre design. Given limited time pre-/post-intervention and number of patients included, a multivariate regression analysis to account for confounders was not performed. Clinical outcomes, impact of one element as compared the whole bundle, and impact on workflow were not assessed. Similar cation interactions exist with tetracyclines and integrase inhibitors and were not addressed. We used time periods pre- and post-intervention that may have been affected by respiratory season, however, pneumonia was the indication for FQ use in a minority of patients.

### Conclusion

The incidence of multivalent cation–FQ DDIs was reduced, but not eliminated, following a bundle of interventions. Future interventions should examine ways to further decrease the incidence of this interaction.

## Supplementary Material

dlag037_Supplementary_Data

## Supplementary data

Figures S1 and S2 are available as Supplementary data at *JAC-AMR* Online.
