# Supplementary material for: In a bind: a quality improvement project for reducing fluoroquinolones drug–drug interactions with multivalent cations
Source: JAC Antimicrob Resist. 2026 Mar 25;8(2):dlag037. doi: 10.1093/jacamr/dlag037 (PMC13014070; doi:10.1093/jacamr/dlag037)
Supplement: dlag037_Supplementary_Data [file dlag037_supplementary_data.docx]

Supplementary materials


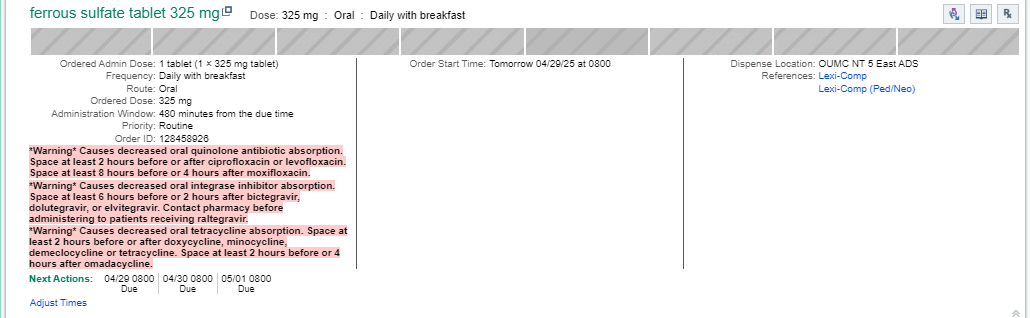


Figure S1- MAR comment on ferrous sulfate


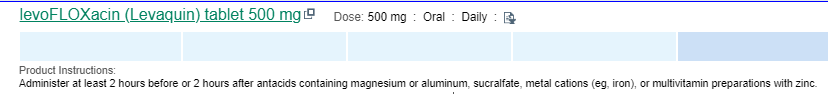


Figure S2 MAR comment on levofloxacin
